# Complete Genome Sequences of Cluster A Mycobacteriophages Kenmech and Peterson and Cluster F Mycobacteriophage Rockne

**DOI:** 10.1128/mra.00908-22

**Published:** 2022-09-29

**Authors:** Imade Y. Nsa, Ayodeji A. Odunsi, Seun D. Bamisaye, Hauwa J. Kamselem, Shina O. Abidoye, Abisola C. Jegede, John O. Ayodele, University of Lagos SEA-PHAGES Annotators, Tenny O. G. Egwuatu, Ganiyu O. Oyetibo, Matthew O. Ilori

**Affiliations:** a Department of Microbiology, Faculty of Science, University of Lagos, Lagos, Nigeria; b Institute of Maritime Studies, University of Lagos, Lagos, Nigeria; DOE Joint Genome Institute

## Abstract

Kenmech, Peterson, and Rockne are bacteriophages that infect Mycobacterium smegmatis mc^2^ 155. Both Kenmech and Peterson genomes are ~52 kbp long and contain 1 tRNA as well as 92 and 89 protein-coding genes, respectively. Rockne has a 56,704-bp genome with 108 protein-coding genes and no tRNA.

## ANNOUNCEMENT

Mycobacterium smegmatis has served as a model organism for studying pathogenic *Mycobacteria*. Recently, bacteriophages isolated on Mycobacterium smegmatis have been used as a therapeutic to treat mycobacterial infections (1–3). The isolation and characterization of novel mycobacteriophages can therefore serve to increase our understanding of their genetic diversity as well as the repertoire of candidate phages for therapeutic use.

Here, we report on mycobacteriophages Peterson and Rockne that were isolated from garden soil in Hermit, PA, and South Euclid, OH, respectively, in 2012, and Kenmech from compost-amended soil in Pittsburgh, PA, in 2020 (Table 1), using standard methods (https://seaphagesphagediscoveryguide.helpdocsonline.com/home, last accessed 15 September 2022). 7H9 liquid medium was added to soil samples and then recovered by centrifugation and filtration (0.02-μm pore size). The filtrate was either plated in top agar with Mycobacterium smegmatis mc^2^ 155, yielding phages Peterson and Kenmech, or first inoculated with Mycobacterium smegmatis mc^2^ 155 and incubated with shaking for approximately 48 h at 37°C before being filtered and plated in top agar containing Mycobacterium smegmatis to yield phage Rockne. All phages were purified through three rounds of plating.

**TABLE 1:** Genome report and features of mycobacteriophages Kenmech, Peterson, And Rockne

Phage	Soil collection site (GPS^*a*^ coordinates)	No. of reads	Sequencing coverage (×)	Genome length (bp)	GC content (%)	Character of genome ends	Genome features		
							No. of predicted protein-coding genes	No. of genes with functional assignments	No. of tRNA genes
Kenmech	40.40782 N, 79.976976 W	80,953	227	52,470	63.7	3′ Sticky overhang	92	45	1
Peterson	41.243272 N, 80.398625 W	588,016	1,535	52,386	63.7	3′ Sticky overhang	89	41	1
Rockne	41.518166 N, 81.527814 W	440,492	1,060	56,704	61.4	3′ Sticky overhang	108	45	0

a GPS, Global Positioning System.

Phage genomic DNA was extracted using the Promega Wizard DNA cleanup kit, prepared for sequencing using the Ultra II library kit v3 (New England BioLabs [NEB]), and sequenced using an Illumina MiSeq (v3 reagents) to yield 150-bp single-end reads. Genome sequences were assembled and checked for completeness using Newbler v2.9 (4) and Consed v29 (5), respectively. Sequencing details and genome characteristics are presented in Table 1.

Automated annotations were performed using DNA Master v5.23.6 (http://cobamide2.bio.pitt.edu [6]), Glimmer v3.02 (7), and GeneMark v2.5 (8), and start sites were refined manually using Starterator v463 (http://phages.wustl.edu/starterator). The putative protein functions were assigned using BLASTp searches against the NCBI nonredundant database v2.13.0 (9) and the actinobacteriophages databases (http://phagesDB.org [10]), Phamerator (11), and HHPred (12). ARAGORN v1.2.41 (13) and tRNAscan-SE v2.0.9 (14) were used for tRNA and transfer-messenger RNA (tmRNA) detection, while transmembrane domains were detected using SOSUI v1.1 (15–17) and TMHMM v2.0 (18, 19). Default parameters were used for all software. Using the Gene Content Similarity (GCS) tool at the Actinobacteriophage database (https://phagesDB.org/genecontent) and based on a GCS of at least 35% to phages in the Actinobacteriophage, Kenmech and Peterson are assigned to subcluster A1 and Rockne to subcluster F1 (10, 20).

Peterson and Kenmech share homologs of several putative genes, including tape measure, DNA primase, scaffolding protein, major tail protein, and a minor tail protein, whereas all three phages encode homologs of terminase large subunit and lysin B (gp13 and gp14 in Peterson).

All three pages encode an immunity repressor and integrase and are therefore predicted to be temperate. Rockne encodes a tyrosine integrase, whereas Peterson and Kenmech encode serine integrases. Excise could be identified for Rockne (gp48) but not for either Peterson or Kenmech. Kenmech and Peterson encode a tRNA-Trp and tRNA-Phe, respectively, whereas Rockne encodes no tRNA as is characteristic of subcluster F1 phages (21).

### Data availability.

This whole-genome shotgun project has been deposited at DDBJ/ENA/GenBank under the accession numbers OP021677 and SRX14483229 for Kenmech, ON970586 and SRX14483243 for Peterson, and ON970595 and SRX14483232 for Rockne. The versions described in this paper are the first versions.
